# No evidence of worsening Arctic springtime ozone losses over the 21st century

**DOI:** 10.1038/s41467-023-37134-3

**Published:** 2023-03-24

**Authors:** L. M. Polvani, J. Keeble, A. Banerjee, R. Checa-Garcia, G. Chiodo, H. E. Rieder, K. H. Rosenlof

**Affiliations:** 1 Department of Applied Physics and Applied Mathematics, Columbia University, New York, NY USA; 2 Lamont-Doherty Earth Observatory, Columbia University, Palisades, NY USA; 3 Department of Chemistry, University of Cambridge, Cambridge, UK; 4 National Centre for Atmospheric Science (NCAS), University of Cambridge, Cambridge, UK; 5 Cooperative Institute for Research in Environmental Sciences, University of Colorado, Boulder, CO USA; 6 Chemical Sciences Laboratory, National Oceanic and Atmospheric Administration, Boulder, CO USA; 7 Institute of Meteorology and Climatology, University of Natural Resources and Life Sciences, Vienna, Austria; 8 Department of Environmental Systems Science, Institute for Atmospheric and Climate Science, ETH Zurich, Zurich, Switzerland

**Keywords:** Projection and prediction, Atmospheric chemistry

**arising from** P. von der Gathen et al. *Nature Communications* 10.1038/s41467-021-24089-6 (2021)

The Montreal Protocol, now ratified by all 198 members of the United Nations, has proven highly effective at mitigating ozone loss and protecting the ozone later. However, a recent paper^1^ in this journal claims that large Arctic springtime ozone losses—driven by chemical processes—could persist or even worsen until the end of this century as a consequence of increasing levels of carbon dioxide. Here we show that such a claim is at odds with the extant literature on the subject, including the latest and most sophisticated modelling studies, which robustly indicate how increased levels of carbon dioxide cause higher, not lower, ozone levels. Hence the alarmist message of that paper is inconsistent with the current understanding of past and future ozone trends.

Recall that the ozone layer protects the Earth’s surface from harmful UV radiation^2^. Hence, not long after the discovery of the ozone hole over Antarctica^3^, the Montreal Protocol was implemented to regulate the production and usage of ozone-depleting substances (ODS). As a consequence, ODS concentrations have started to decrease in the late twentieth century^4,5^, and are expected to continue decreasing over the twenty-first century^6^. The healing of the ozone layer over Antarctica has already been reported^7^.

While stratospheric ozone depletion over the Arctic has been much smaller than over the Antarctic^8^, every few years low ozone concentrations are observed at high northern latitudes^9–11^. Stratospheric ozone losses in the polar regions result from the presence of polar stratospheric clouds (PSC)^12^, which allow for the formation of the highly reactive chlorine species directly responsible for ozone loss^13^. From PSC estimates, a claim was made that Arctic ozone minima in the last several decades have been getting worse as a consequence of “climate change”^14,15^, but that claim has been disputed^16–18^, and it was clearly shown that Arctic ozone minima in recent decades are not related to increased levels of carbon dioxide, but to the presence of ODS^19^.

It comes as a surprise, therefore, that a recent paper^1^ in this journal (hereafter VDG) carries the title “Climate change favours large seasonal loss of Arctic ozone.” In the last sentence of the paper, summing up the key findings, we read that “anthropogenic climate change has the potential to partially counteract the positive effects of the Montreal Protocol in protecting the Arctic ozone layer.” Taken at face value, such a statement means that increasing levels of carbon dioxide will be damaging to the ozone layer, specifically in causing deeper ozone minima over the Arctic in springtime. Even more, it suggests that the Montreal Protocol may no longer be sufficient in protecting the ozone layer in the presence of accelerating climate change. If this were the case, the findings of this paper would be the cause of much alarm.

However, a careful reading of VDG reveals that the scientific findings of that paper are actually much narrower than the sentence we have just cited claims. Specifically, the paper is concerned the narrow question of chemical ozone loss in the spring season. While chemical loss is an important process, which needs to be understood in its own right, ozone levels are not controlled by chemistry alone. The atmospheric circulation, in particular, plays a crucial role in determining the levels of stratospheric ozone. When all the relevant process are included, as they are in the state-of-the-art comprehensive chemistry-climate models, there is no evidence that future ozone levels will decrease in the coming decades, including over the Arctic in springtime, as we now explicitly show.

First, let us look at some of the very latest model projections. In Fig. 1 (left panels), we have plotted the total ozone column in March, averaged over the Arctic, from 1950 to 2100, for five models participating in the recent Coupled Model Intercomparison Project, Phase 6 (CMIP6)^20^. Unlike most CMIP6 models in which ozone levels are prescribed a priori, these five models include interactive chemistry schemes that actually compute the ozone concentrations from chemical reactions consistently with the time evolution of all natural and anthropogenic emissions, and with each model’s temperature, moisture, PSC surface area, and atmospheric circulation. In other words, these models incorporate all the important processes that affect ozone levels, not just chemical loss. We emphasise that the Arctic ozone column in these models are consistent with the observed ozone column over the period 1979-2021, as one can see in Fig. 1 (panels a and c).

**Fig. 1:**
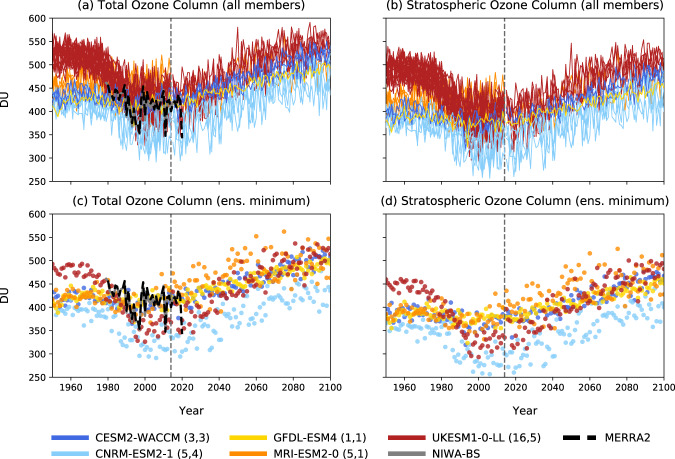
Arctic column ozone in March, in five CMIP6 models under SSP5-8.5, and in observations. The top panels (**a**, **b**) show all the members of our multi-model ensemble, and the bottom panels (**c**, **d**) the minimum over that ensemble. The left panels (**a**, **c**) show the total ozone column, the right panels (**b**, **d**) the stratospheric column alone. For the period 1950–2014 we have plotted the historical simulations; for the period 2014–2100, the SSP5-8.5 simulations. The model names are given in the legend, followed by the corresponding number of simulations shown for the historical and SSP5-8.5 portions, respectively, in parentheses. The solid grey and dashed black lines show the NIWA-BS^27^ and MERRA2^28^ reanalyses, respectively. All ozone columns are here averaged over the Arctic, defined as the region 60^∘^−90^∘^N.

The key point of that figure is that, in these five model, the total ozone column in March shows no evidence of decreasing over the twenty-first century, indicating that any potential chemical losses are overwhelmed by other processes. In particular, every ensemble member of every model shows a larger total ozone column in the future than at present (panel a), and the same applies to the ensemble minimum (panel b). And, as seen in the right panels, the total ozone column is dominated by stratospheric ozone, whose concentrations increase in the coming decades with the waning of ODS. We emphasise that Fig. 1 shows the SSP5-8.5 simulations, the scenario with the largest CO_2_ increases among the current emission scenarios. Clearly, large and sustained emissions of CO_2_ are not accompanied by large Arctic ozone depletion in this scenario.

Second, and most importantly, the ozone projections of the models in Fig. 1 are in no way surprising or exceptional. Those models simply confirm the findings of a long series of multi-model and single-model studies, with progressively more sophisticated chemistry-climate models, that have unfailingly shown that ozone levels will increase in the coming decades. Just to cite the multi-model literature, starting from the most recent: “ozone recovery,” as it is called, has been reported—over the Arctic in springtime—by the CMIP6 models (Fig. 7 of ref. ^20^), by the Chemistry-Climate Model Initiative (CCMI) models (Fig. 3 of ref. ^21^), by the CMIP5 models (Fig. 6 of ref. ^22^), by the Chemistry-Climate Model Validation project, Phase 2 (CCVal-2) models (Fig. 6 of ref. ^23^), and by the earlier CCMVal models (Fig. 8 of ref. ^24^). The full list of peer-review papers showing that ozone levels will increase over the 21st Century is much longer, and goes further back in time, but these references should suffice.

Third, it is important to recall that all these studies have also consistently shown that future ozone levels—over the Arctic in March—will be higher for the higher emission scenarios: this is clearly shown, for example in Fig. 2e of ref. ^25^. The claim in the abstract of VDG, that “conditions favourable for large, seasonal loss of Arctic column ozone could persist or even worsen until the end of this century, if future abundances of GHGs continue to steeply rise”, which is based on an proxy index of future loss, not the actual model output of ozone loss, appears to contradict a large body of evidence from chemistry-climate models, which have consistently shown how rising GHG will lead to higher ozone levels, not the other way.

Finally, regarding the narrow question of seasonal chemical ozone loss in coming decades: we do not understand why VDG decided to construct a complex ozone loss potential (OLP) proxy index, which involves many assumptions and free parameters, instead of simply examining the actual chemical loss in chemistry-climate models such as those in Fig. 1, which are perfectly capable of simulating the  observed ozone column over the Arctic in springtime. One would have expected VDG to validate their OLP index by comparing its predictions against actual chemical ozone loss in such chemistry-climate models. Needless to say, a careful analysis of the complex OLP index in VDG is beyond the scope of this brief comment. We here limit ourselves to two observations.

First, contrasting Figs. 8 and 9 in VDG, one can see that their claim of future seasonal chemical ozone loss rests crucially on the stratospheric water vapour used to construct the OLP index. And yet, among the very many assumptions underlying their OLP computation, VDG opted not use the actual water vapour as computed in each model. Rather, they employed a simple empirical formula meant to account for the effect of methane oxidation on stratospheric water vapour, so that the water vapour in the OLP index is inconsistent with each model’s  own water vapour.

Second, we note a more fundamental inconsistency in VDG’s methodology. Their OLP index is based, largely, on stratospheric temperatures which, obviously, are linked to ozone levels. Recalling that in the majority of CMIP6 models ozone levels are prescribed, not computed, one wonders: how can projected temperatures from models in which ozone is prescribed be used to make projections about ozone itself? This leads to an apparent contradiction: the ozone levels prescribed in most CMIP6 models increase over the twenty-first century^20^, and yet those same models are telling us that large future seasonal chemical ozone losses “will persists or even worsen until the end of this century,” if we are to believe VDG. It has been shown that current chemistry-climate models—in which ozone levels are computed consistently with each model’s temperature, circulation, and anthropogenic emissions—are able to capture the observed trends in stratospheric temperatures^26^. If, therefore, one felt the need to know whether future seasonal chemical ozone losses will differ from present ones, examining the model output from chemistry-climate models would be superior to constructing an inconsistent proxy OLP index.

In any case, the key point we would like to emphasise is that chemical ozone loss is only one part of the ozone story. If one is concerned about societally relevant impacts, the actual ozone concentration (not just the chemical loss) is what really matters. We reiterate that chemistry-climate models robustly project that stratospheric ozone concentrations will increase in the future, both globally and over the Arctic, and that they will increase more in the presence of higher levels of CO_2_ (this has been termed “super-recovery”). And, it should be abundantly clear, the cause of this future ozone increase is the continued reduction of ODS thanks to the Montreal Protocol, signed in 1987 and now ratified by every single member country of the United Nations.

## Data Availability

Model output for the five CMIP6 models shown in this paper is available from the World Climate Research Programme at https://www.wcrp-climate.org/wgcm-cmip/wgcm-cmip6 (last access: Nov 2022). The MERRA2 ozone reanalysis data are available from the Goddard Earth Sciences Data and Information Services Center at https://disc.gsfc.nasa.gov/datasets?project=MERRA-2 (last access: Nov 2022). Version 3.4 of the National Institute of Water and Atmospheric Research-Bodeker Scientific (NIWA-BS) combined TCO database is available at http://www.bodekerscientific.com/data/total-column-ozone (last access: Nov 2022).
